# Improved Endovascular Aortic Repair Durability in Patients Achieving Increased Shortest Apposition Length: A Multi-Centre Analysis

**DOI:** 10.1177/15266028251338812

**Published:** 2025-05-13

**Authors:** Cas H. F. Hendricks, Richte C. L. Schuurmann, Bram Fioole, Rogier H. J. Kropman, Reinoud P. H. Bokkers, Lievay van Dam, Jan-Albert Vos, Jean-Paul P. M. de Vries

**Affiliations:** 1 Division of Vascular Surgery, Department of Surgery, University Medical Centre Groningen, Groningen, the Netherlands; 2 Division of Vascular Surgery, Department of Surgery, Maasstad Hospital, Rotterdam, the Netherlands; 3 Division of Vascular Surgery, Department of Surgery, St. Antonius Hospital, Nieuwegein, the Netherlands; 4 Division of Interventional Radiology, Department of Radiology, University Medical Centre Groningen, Groningen, the Netherlands; 5 Division of Interventional Radiology, Department of Radiology, Maasstad Hospital, Rotterdam, the Netherlands; 6 Division of Interventional Radiology, Department of Radiology, St. Antonius Hospital, Nieuwegein, the Netherlands

**Keywords:** abdominal aortic aneurysm, endovascular aneurysm repair, endoleak, seal, shortest apposition length

## Abstract

**Objective:**

Endovascular aortic repair (EVAR) for an aneurysm of the abdominal aorta (AAA) is associated with long-term complications, such as endoleaks, resulting in a significant re-intervention rate. This study investigates the prognostic value of (change of) proximal seal length on post-EVAR computed tomography angiography (CTA) for predicting type 1a endoleak. It further proposes a risk-stratified imaging follow-up algorithm.

**Design:**

Multicentre, retrospective, observational study of consecutive patients who underwent elective EVAR for infrarenal AAA between 2015 and 2018 at 3 high-volume hospitals in the Netherlands.

**Materials and Methods:**

Aorta morphology and endograft position analysis was performed. Shortest apposition length (SAL) was measured on the first post-EVAR CTA and, if available, on the last CTA. Change of SAL through time was categorized as increasing, stable, or decreasing and correlated with type 1a endoleak and secondary interventions for endoleak. Kaplan–Meier analysis was used to calculate type 1a endoleak free and re-intervention-free survival.

**Results:**

Three hundred ten AAA patients with a median follow-up of 51 (Q1, 17; Q3, 71) months were included. A median SAL of 22.8 mm (Q1, 15.9; Q1, 30.4) was measured on the first post-EVAR CTA. In 168 of 310 patients (54%), a second post-EVAR CTA was available, in which 71 (42%) showed increasing SAL over time. No type 1a endoleak developed in the increasing SAL group, whereas 1 of 43 (2%) in the stable group and 10 of 54 (19%) in the decreasing group developed type 1a endoleak. Five years post-EVAR, type 1a endoleak-free survival was 100% in the increasing SAL group versus 97.1% in the stable SAL group (p=0.195), and 81.6% in the decreasing SAL group (p<0.001). The re-intervention for all types of endoleak-free survival was 100% in the increasing SAL group versus 84.6% in the stable SAL group (p<0.001), and 60.7% (p<0.001) in the decreasing SAL group.

**Conclusion:**

Increasing SAL after EVAR for infrarenal degenerative AAA is an indicator of durable success without type 1a endoleak and endoleak-associated secondary intervention within 5 years. Decreasing SAL is associated with development of type 1a endoleak after EVAR. Evaluation of (change of) the proximal seal could be a valuable part of follow-up after EVAR.

**Clinical Impact:**

Evaluation of proximal seal length after endovascular aortic repair offers valuable prognostic information regarding the risk of type 1a endoleak. Implementation could refine current follow-up algorithms to better stratify patients who have a substantial risk of type Ia endoleak from patients who may benefit from limited image surveillance.

## Introduction

Endovascular aortic repair (EVAR) is now considered to be first choice of treatment for patients with an infrarenal aneurysm of the abdominal aorta (AAA), but long-term follow-up is associated with endoleaks, migration, and limb occlusion, resulting in a substantial re-intervention rate.^1^ A recent meta-analysis reports a 5-year re-intervention rate of 19%.^2^ This is often due to asymptomatic, high-risk graft-related complications such as type 1a endoleak.^3^ Regular imaging surveillance after treatment is therefore required. The current Society of Vascular Surgery practice guidelines on the care of patients with an AAA recommend computed tomography angiography (CTA) imaging at 1 month and 1 year post-EVAR.^4^ An additional CTA at 6 months is recommended if a type 2 endoleak is observed at 1 month. After that, annual imaging using duplex ultrasound is recommended. The disadvantages of this surveillance strategy are high costs, the demand for hospital and outpatient capacity, exposure to ionizing radiation, and the psychological burden for the patient.^5^ Implementing a risk-stratified imaging surveillance approach after EVAR, based on preoperative aortic anatomy and postoperative CTA findings, could help mitigate these drawbacks.^6,7^

Geraedts et al showed that the risk of developing late type 1a endoleak was significantly higher in patients with <10 mm proximal seal at the first post-EVAR CTA.^8^ In addition, diminishing seal length seen in subsequent follow-up scans was also a strong predictor of late type 1a endoleak.^9^

The current study aims to further investigate the prognostic value of (change of) proximal seal length on post-EVAR CTA on type 1a endoleak in a consecutive cohort of patients who underwent elective EVAR. This study also aims to propose a follow-up algorithm to stratify patients who have a substantial risk of type 1a endoleak post-EVAR from patients who might benefit from limited image surveillance.

## Materials/Patients and Methods

### Study Design and Inclusion and Exclusion Criteria

The current study is a multicentre, retrospective, observational study of consecutive patients who underwent elective EVAR for a degenerative infrarenal AAA between 2015 and 2018 at 3 high-volume EVAR hospitals in the Netherlands (University Medical Centre Groningen, Maasstad Hospital Rotterdam, and St. Antonius Hospital Nieuwegein). Patients treated with a nonfenestrated abdominal endograft from manufacturers Cook (Cook Medical, Bloomington, IN, USA), Medtronic (Medtronic, Inc., Minneapolis, MN, USA), Gore (W. L. Gore & Associates Inc., Flagstaff, AZ, USA), and Bolton Medical (Bolton Medical, Inc., Sunrise, FL, USA) were included. A pre-EVAR CTA had to be available as well as at least 1 postoperative CTA within 3 months post-EVAR. Patients for whom additional proximal fixation was performed during EVAR such as EndoAnchors (Medtronic Vascular, Santa Rosa, CA, USA) were excluded from this study.

### Data Collection

Patient demographics were collected and stored using the electronic data capture program REDCap (Vanderbilt University, Nashville, TN, USA). For each patient, the last pre-EVAR and the first post-EVAR CTA scan were obtained. If multiple postoperative CTA scans were available, the last postoperative CTA scan (at least 10 months post-EVAR) was also obtained so that the difference in aneurysm diameter and proximal seal could be calculated. If a patient developed a type 1a endoleak or if a secondary intervention was performed to improve the proximal seal, the CTA prior to this event was obtained to calculate the differences in aneurysm diameter and proximal seal.

### Follow-Up Protocols

In each of the 3 participating centers, a preoperative CTA and an early postoperative CTA (typically within 6–8 weeks) were considered standard of care. If the first postoperative CTA showed no endoleak or graft-related complications, yearly follow-up was generally conducted with duplex ultrasonography (DUS); however, follow-up imaging method, timing, and intensity could be adjusted at the discretion of the treating physician. If DUS showed an endoleak, aneurysm growth (≥5 mm), or other graft-related complications, a CTA was made. CTA scans were assessed and validated by an experienced certified radiologist. DUS imaging was performed by experienced certified vascular sonographers. Presence of post-EVAR complications (endoleak, aneurysm growth) was extracted from radiologic and sonographic reports. The total follow-up time of patients was set at the date of the last available CTA or DUS.

### Measurement Protocol

Aorta morphology and endograft position analysis was done using the vascular workstation 3mensio 10.1 (Pie Medical Imaging BV, Maastricht, the Netherlands) and postprocessing software Vascular Imaging Analysis (Endovascular Diagnostics BV, Bussum, the Netherlands). The measurement protocol has been published and verified before by Schuurmann et al.^10^ Measurements were performed by an experienced researcher and verified by a second researcher if needed. Maximum aneurysm diameter was measured outer-to-outer wall. Intended over-sizing was defined as (nominal proximal endograft diameter/aortic neck diameter − 1) × 100%. Whether the primary endograft placement was used within the instructions for use (IFU) was determined for each patient, focusing on the aortic neck criteria.

**Figure 1.**
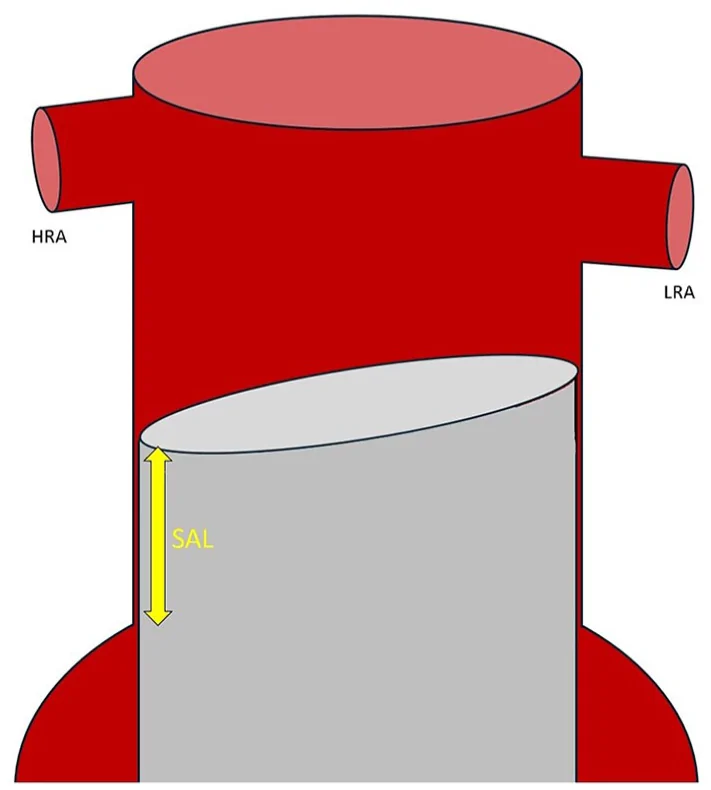
Schematic drawing portraying SAL; the shortest distance along the aortic wall between the start of the proximal endograft fabric and the point where circumferential apposition with the aortic wall was lost. HRA, highest renal artery; LRA, lowest renal artery; SAL, shortest apposition length.

The VIA software was used to measure the shortest apposition length (SAL) in all postoperative CTA scans. As shown in Figure 1, SAL was defined as the shortest distance along the aortic wall between the start of the proximal endograft fabric and the point where circumferential apposition with the aortic wall was lost. For patients with multiple postoperative CTA scans available, the difference in SAL and maximum aneurysm diameter between the first and the last postoperative CTA was calculated. A ≥5 mm growth or shrinkage was considered an increase or decrease in maximum aneurysm diameter, respectively. A change of <5 mm in maximum aneurysm diameter was considered stable. Schuurmann et al reported a repeatability coefficient for SAL measurements of 4.1 mm at a 95% confidence interval.^10^ A ≥2 mm increase or decrease of SAL was considered increasing or decreasing SAL, respectively. A <2 mm change of SAL was considered stable.

### Statistical Analysis

Statistical analysis was performed using IBM SPSS statistical analysis software (IBM Corp. Released 2021. IBM SPSS Statistics for Windows, Version 29.0; IBM Corp., Armonk, NY, USA). Normal distribution of continuous data was assessed by visual inspection, quantile–quantile plots and a Shapiro–Wilk test. Normally distributed data are expressed as means with the standard deviation, and nonnormally distributed data are expressed as medians with the first and third quartile. Differences in categorical data were tested using the χ^2^ test or Fisher exact test. Differences in continuous data were assessed with a 2-samples *t* test for normally distributed data and a nonparametric Mann–Whitney *U* test for nonnormally distributed data. Spearman’s correlation test was used to assess the correlation between change of SAL over time and sac dynamics. Kaplan–Meier survival analysis was performed to determine type 1a endoleak-free survival as well as re-intervention for endoleak (all types) free survival. Differences in these survival curves were tested using the Log Rank test. Survival curves were truncated when the standard error of the patency rate estimate is >10%. A p value of ≤0.05 was considered statistically significant.

### Ethical Considerations

This study was conducted in compliance with the Declaration of Helsinki. The protocol was approved by the central ethics review board non-WMO studies of the University Medical Centre Groningen under research register number 17626. It was subsequently approved by local medical ethics committees at the other 2 hospitals.

## Results

### Inclusions

**Figure 2.**
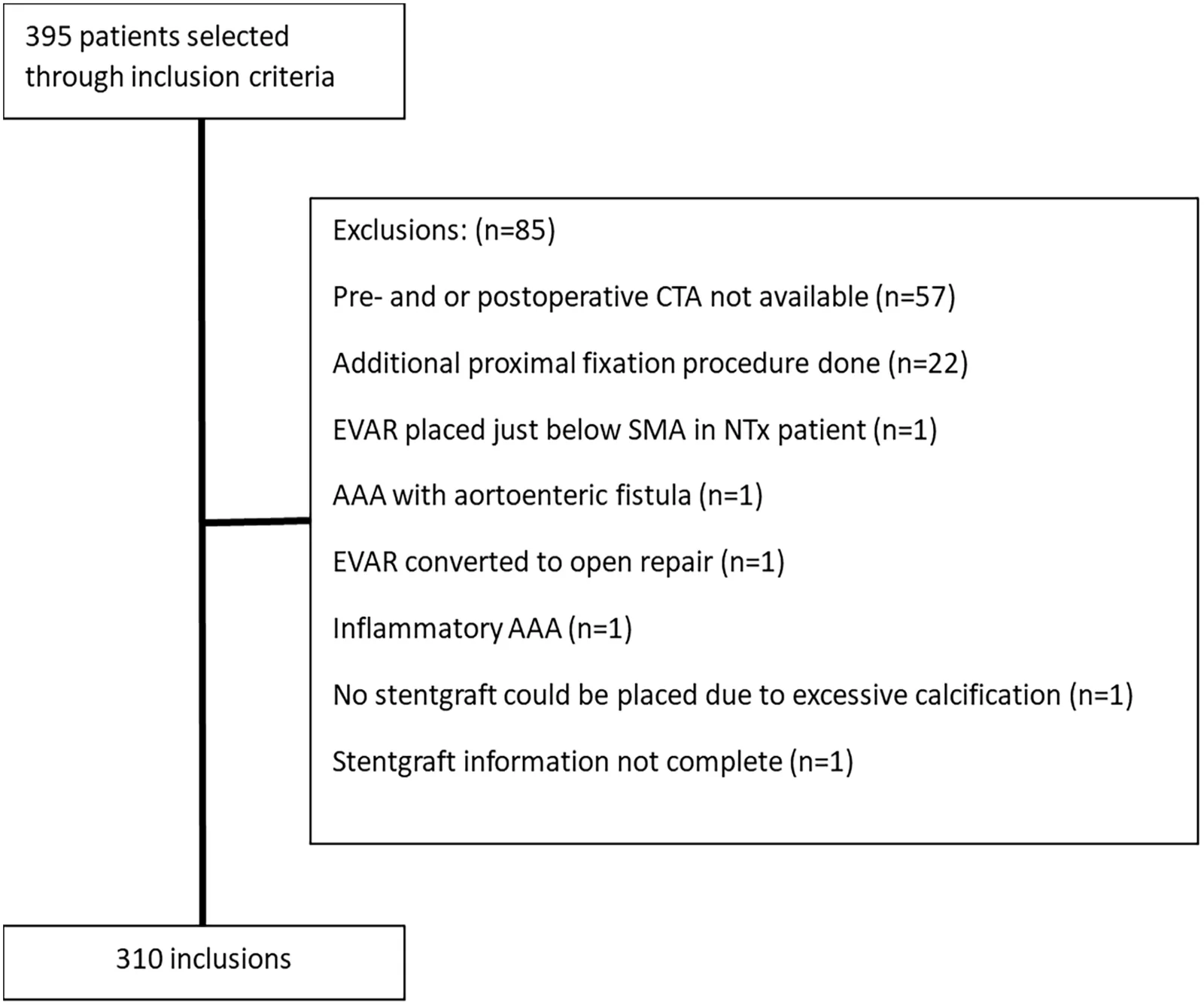
Inclusion process. AAA, abdominal aortic aneurysm; CTA, computed tomography angiography; EVAR, endovascular aortic repair; NTx, kidney transplant; SMA, superior mesenteric artery.

From January 2015 to December 2018, 395 patients were treated electively for a degenerative infrarenal AAA by EVAR in the 3 participating hospitals. After the exclusion criteria were applied, 310 patients were included in this study (Figure 2). Baseline characteristics are displayed in Table 1.

**Table 1.** Baseline Patient Characteristics.

Variable	Value^a^, n=310
Age, years	73 ± 7.8
Male, gender, *n* (%)	273 (88)
ASA classification, *n* (%)	
ASA 1 or 2	162 (52)
ASA 3	144 (47)
ASA 4	4 (1)
Device, *n* (%)	
Medtronic Endurant (II or IIs)	126 (41)
Cook Zenith (Flex or Alpha)	99 (32)
Gore Excluder	72 (23)
Bolton Medical Treovance	13 (4)
Aorto-uni-iliac configuration, *n* (%)	27 (9)
Nominal proximal endograft diameter, mm	28 (26, 32)
Maximum aneurysm diameter, mm	59.0 (55.3, 63.3)
Intended oversizing, %	20 (14, 25)
Neck characteristics	
Neck diameter, mm	23.8 (22.0, 25.4)
Neck length, mm	28 (20, 41)
Suprarenal angulation, degrees	17 (10, 25)
Infrarenal angulation, degrees	44 (34, 57)
Not within IFU, *n* (%)	79 (25)
Reason not within IFU, *n* (%)	
Neck diameter	4 (5)
Neck length	19 (24)
Infrarenal angulation	34 (43)
Suprarenal angulation	3 (4)
Neck diameter and infrarenal angulation	2 (2)
Neck length and infrarenal angulation	6 (8)
Supra- and infrarenal angulation	11 (14)

Abbreviations: ASA, American Society of Anesthesiologists; IFU, instructions for use.

a Normally distributed continuous data are presented as the means ± standard deviation; nonnormally distributed continuous data are presented as the means with first and third quartile; categorical data are given as the counts (percentage).

### Early Postoperative CTA Results

For the entire cohort, a median SAL of 22.8 mm (15.9, 30.4) was measured on the first postoperative CTA. In 44 of 310 patients (14%), the SAL was <10 mm on the first postoperative CTA. Three patients showed a type 1a endoleak on the first postoperative CTA. None of these type 1a endoleaks were seen on the intraoperative completion angiogram. Two of these 3 patients were subsequently treated by proximal nonfenestrated extension. For the third patient, the risk of a secondary intervention was deemed too high due to advanced age and presence of multiple comorbidities, and thus best supportive care was provided. The first postoperative CTA in 73 of 310 patients (24%) showed a type 2 endoleak. Six of these patients were eventually treated by embolization due to aneurysm growth (≥5 mm) during follow-up.

### Mid-Term and Long-Term Follow-Up Results

The median total follow-up time was 51 (17, 71) months. In 168 of 310 patients (54%), a second CTA at least 10 months post-EVAR was available for analysis. The median time between EVAR and the latest available follow-up CTA was 40 (20, 63) months. For these patients, a difference in SAL was calculated between the first post-EVAR CTA and the last CTA. In 71 of 168 (42%) of these patients the SAL had increased on the last follow-up CTA, with a median of 7.3 (3.8, 14.4) mm over time. In 43 of 168 patients (26%) the SAL was stable. In 54 of 168 (32%), the SAL had decreased with a median of −7.8 (−15.3, −3.8) mm. With regard to the infrarenal neck, 231 of 310 patients (75%) were treated within the endograft manufacturer’s IFU. For patients with a second CTA available, 127 of 168 (76%) were treated within IFU versus 104 of 142 (73%) patients without a second CTA available (p=0.695). In 20 of 168 (12%) patients, the SAL on the first postoperative CTA was <10 mm, versus 24 of 142 (17%; p=0.253).

**Figure 3.**
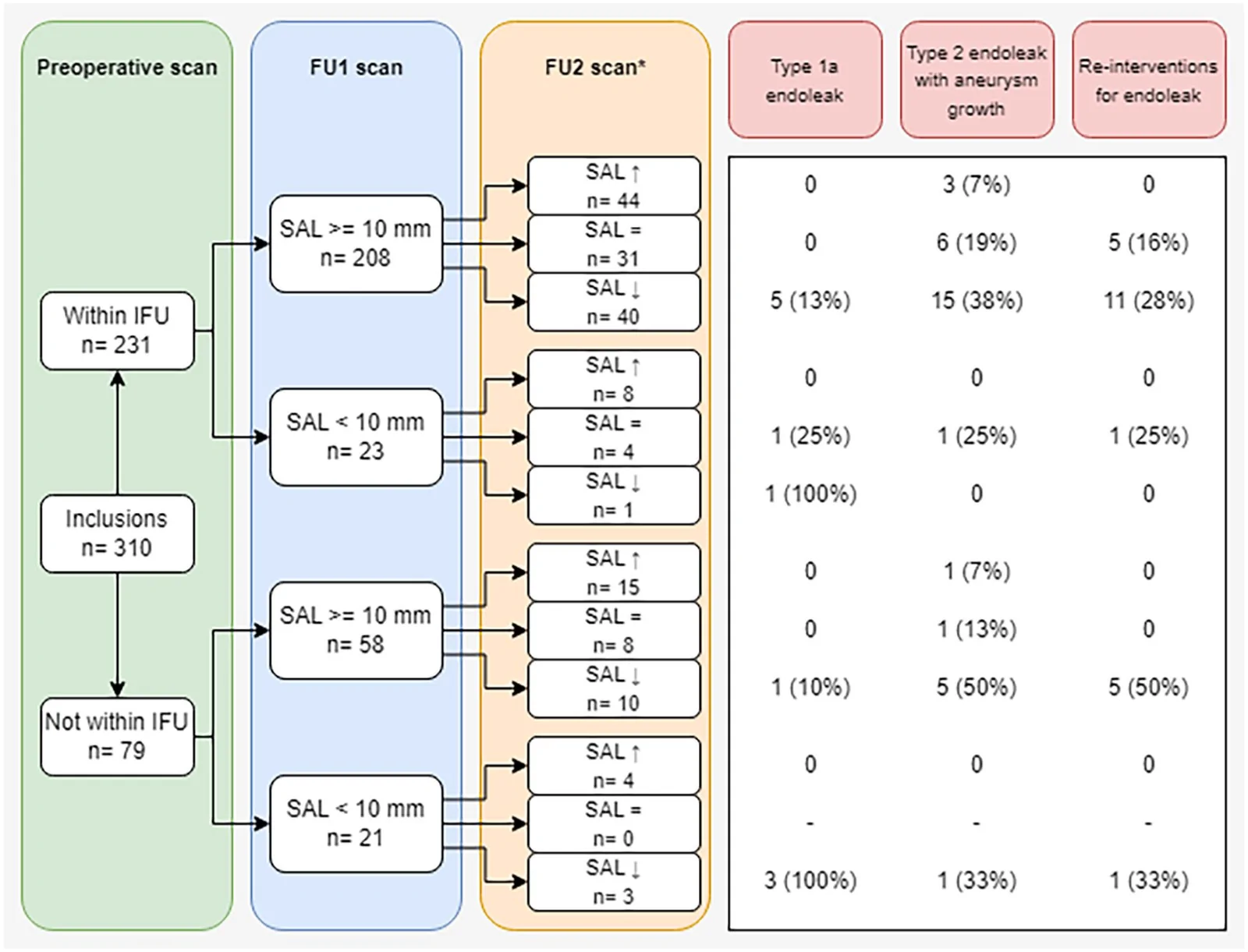
Flowchart depicting the number of complications and re-interventions during follow-up post-EVAR for each group based on IFU status, SAL on FU1 and evolution of SAL over time. EVAR, endovascular aortic repair; FU1, first post-EVAR; FU2, last available post-EVAR; IFU, instructions for use; SAL, shortest apposition length. *Only patients with a second postoperative CTA available were included in this part of the flowchart.

IFU status (inside vs outside), SAL on the first postoperative CTA (<10 mm vs ≥10 mm), and change of SAL over time (increase, stable or decrease) were used to split the total cohort into 12 groups. Figure 3 shows a flowchart depicting the number of complications (type 1a endoleak or type 2 endoleak with aneurysm growth) and re-interventions for endoleak for each group. The median time between EVAR and secondary intervention for endoleak was 41 (23, 55) months.

There were no statistically significant differences between the increasing, stable or decreasing SAL groups in terms of IFU status or endograft device used (p=0.352 and p=0.845, respectively). No significant difference was found between the groups for infrarenal versus suprarenal fixating endografts (p=0.481).

Presence of neck thrombus (≥2 mm thick and ≥25% circumference) and conical neck shape (≥2 mm diameter increase per 1 cm of neck length) was also not statistically significant between groups (p=0.871 and p=0.204, respectively). Presence of neck calcification (≥2 mm thick and ≥25% circumference) was seen in 15 of 54 (28%) patients in the decreasing SAL group, versus 8 of 43 in the stable SAL group, and 8 of 71 in the increasing SAL group (p=0.062).

In 77 of 168 (46%) patients, the maximum aneurysm diameter had decreased over time. In 62 of 168 (37%) patients, it was stable, and in 29 of 168 (17%) patients, the maximum aneurysm diameter had increased over time. Spearman’s correlation test found a weak negative correlation between change of SAL over time and sac dynamics (ρ=−0.179, p=0.020).

### Complications and Re-Interventions of Increasing, Stable, and Decreasing SAL Groups

None of the patients in groups in which SAL increased over time, regardless of IFU-status and SAL on the first postoperative CTA, developed a type 1a endoleak during follow-up. Of these patients, 4 developed type 2 endoleak with aneurysm growth (≥5 mm). None of the patients in this group received a secondary intervention for endoleak during follow-up.

In patients for which SAL remained stable over time, only 1 patient developed a type 1a endoleak, after 35 months post-EVAR. Eight patients (19%) developed type 2 endoleak with aneurysm growth during follow-up. Secondary intervention for endoleak was performed in 6 of 43 patients (14%): 2 distal extensions for type 1b endoleak and 3 embolizations for type 2 endoleak. In 1 patient, open surgical repair was performed for aneurysm growth up to a maximum diameter of 10 cm, without a visible endoleak on the CTA scan. A fabric tear of the endograft was seen intra-operatively (type 3 endoleak), which likely caused the growth.

For patients in the decreasing SAL group, 10 of 54 (19%) developed type 1a endoleak during follow-up, and 21 (39%) developed type 2 endoleak with aneurysm growth. Re-interventions for endoleak were performed in 17 of 54 (31%) of these patients. These included 2 open repairs for type 1a endoleak, 1 proximal extension for type 1a endoleak, 2 FEVAR for diminishing proximal seal, 5 distal extensions for type 1b endoleak, and 7 embolizations for type 2 endoleak. In 5 patients with a type 1a endoleak, the risk of a secondary intervention was deemed too high due to advanced age and presence of multiple comorbidities. In 14 patients with a type 2 endoleak and aneurysm growth, no secondary intervention was performed. This was mainly because the treating physician opted for imaging-based follow-up (in case of limited aneurysm growth), or due to the simultaneous presence of a type 1 endoleak, which was treated first.

### Type 1a Endoleak Free Survival and Re-Intervention for All Types of Endoleak Free Survival

**Figure 4.**
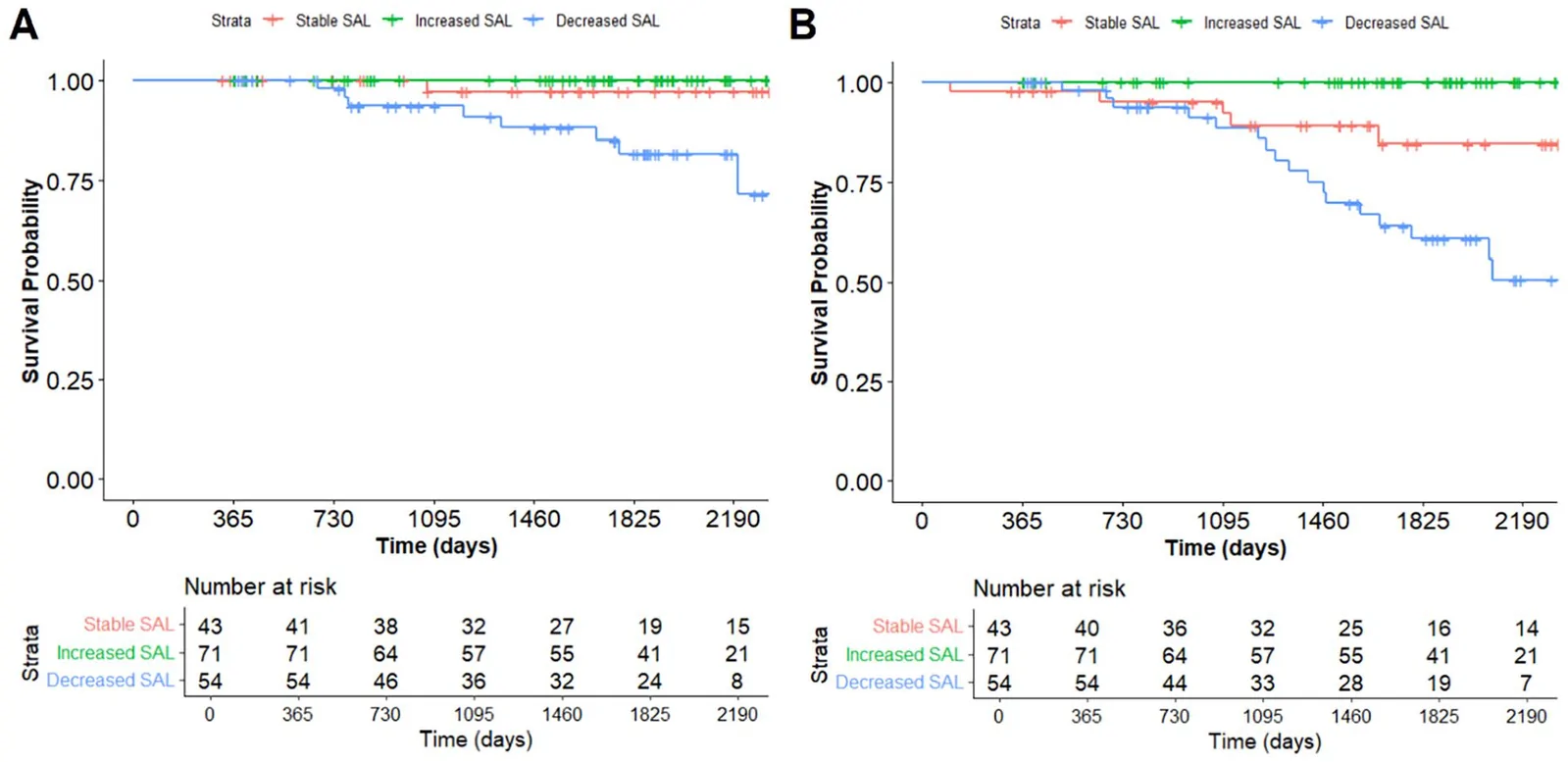
Kaplan–Meier estimates of type 1a endoleak free survival (A) and re-intervention for endoleak-free survival (B).

Five years post-EVAR, type 1a endoleak-free survival was 100% in the increasing SAL group versus 97.1% in the stable SAL group (p=0.195), and 81.6% in the decreasing SAL group (p<0.001) (Figure 4A).

At 5 years post-EVAR, the re-intervention for all types of endoleak-free survival probability was 100% in the increasing SAL group versus 84.6% in the stable SAL group (p<0.001), and 60.7% (p<0.001) in the decreasing SAL group (Figure 4B).

## Discussion

In this study of the prognostic value of (change of) proximal seal length on type 1 endoleak, we found that patients who showed an increase of SAL over time did not develop type 1a during follow-up. Of patients with a stable or decreasing SAL over time, 1 of 43 (2%) and 10 of 54 (19%) developed a type 1a endoleak during follow-up respectively.

Surveillance after EVAR continues to be a subject of discussion. It is regarded as mandatory to monitor for graft-related complications, but on the other hand, evidence of its benefit to survival is lacking.^11^ Add to that the high costs, radiation exposure, and patient burden and there seems to be enough reason to move from a fixed follow-up algorithm to a risk-stratified model based on imaging findings. This is highlighted in the current practice guidelines, which emphasize the need for further research to the most cost-effective and clinically effective surveillance after EVAR.^4^

Performing EVAR outside of endograft’s IFU has been shown to increase the risk of late failure.^12–14^ The same is true for insufficient apposition length (proximal and/or distal of <10 mm) or presence of endoleak on the first postoperative CTA.^15^ Our results correspond with this. However, these preoperative and early postoperative variables might not be discriminative enough for adequate long-term risk stratification in follow-up algorithms. Evaluating variables on subsequent postoperative imaging could make these algorithms more robust. For instance, the current guidelines for management of AAA by the European Society for Vascular Surgery recommend evaluating sac dynamics on annual CTA or DUS.^16^ This is a valid strategy to assess durable EVAR success, although it might not be powerful enough to determine imaging follow-up.^17,18^ This is reflected in our study, as we saw a significant, but weak negative correlation between change of SAL over time and sac dynamics. Aneurysm growth may cause an increase of the distal infrarenal neck diameter, leading to a decrease in SAL, while aneurysm shrinkage could lead to an increase of SAL. However, this relationship is not always consistent. For instance, proximal neck dilatation can compromise the proximal seal even as the aneurysm sac shrinks, potentially necessitating reintervention. Change of seal length over time might be another valuable variable to assess risk of complication or need for secondary intervention.

The current study suggests that decreasing SAL over time is associated with development of type 1a endoleak after EVAR. This is in line with the results of a previous study by Zuidema et al, where they found that patients who developed late type 1a endoleak showed diminishing SAL during follow-up.^9^

**Figure 5.**
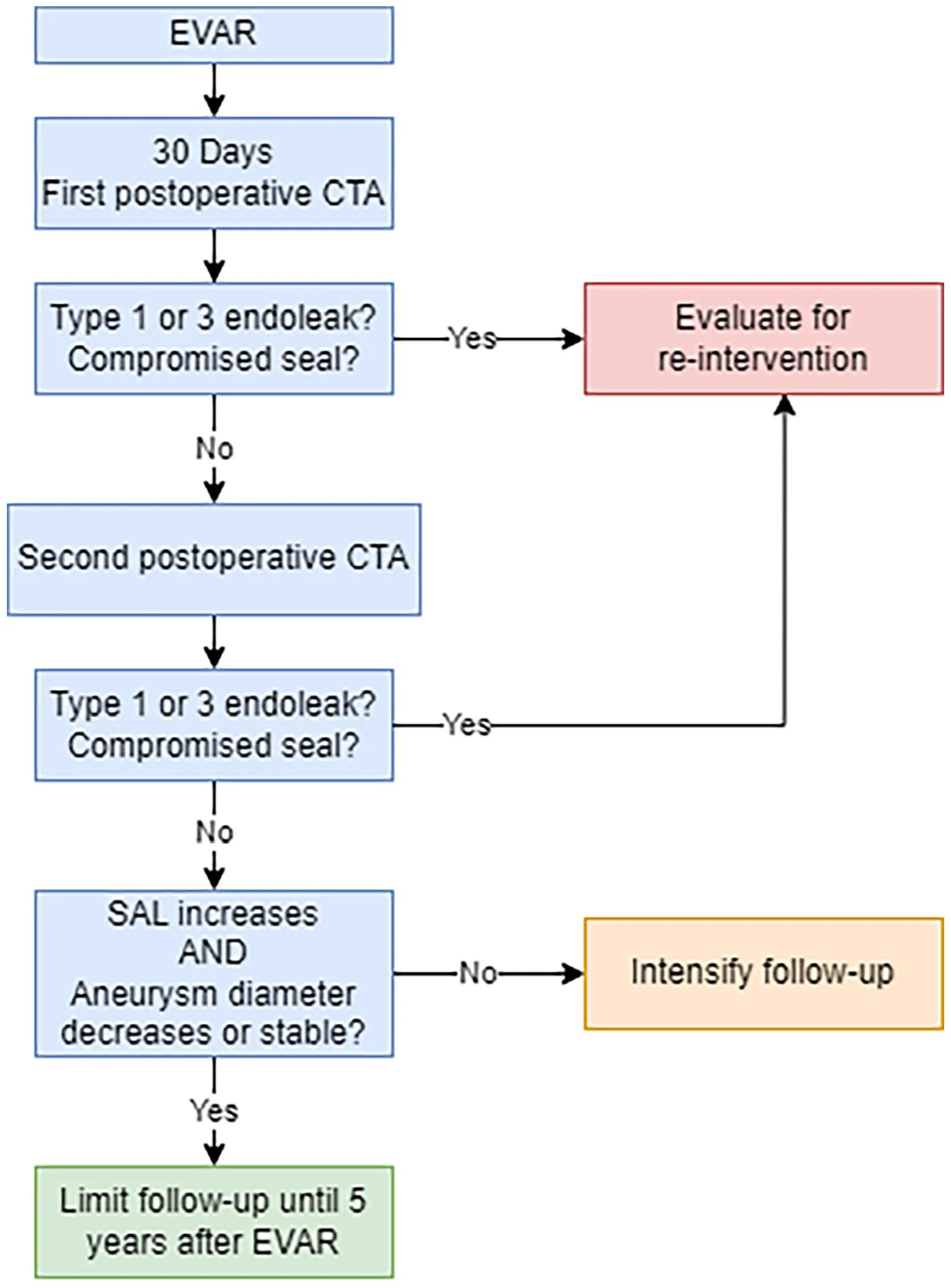
New proposed follow-up algorithm, which includes evaluation of evolution of SAL and aneurysm diameter. CTA, computed tomography angiography; EVAR, endovascular aortic repair; SAL, shortest apposition length.

We propose a follow-up algorithm which incorporates careful assessment of apposition on the first postoperative CT scan and change of apposition on a later CT scan (Figure 5). This means that we suggest that at least 2 postoperative CTA scans are made to evaluate change of SAL over time, because measurement of seal length is not possible on DUS. Based on our data, the timing of the second postoperative CTA should be within 2 years after EVAR, as to identify high-risk patients before a type 1a endoleak develops. In our proposed algorithm, patients who show an increase of SAL and favorable sac dynamic (stable or decreased maximum aneurysm diameter) on the second postoperative CTA are at a low risk of endoleak. We propose limiting imaging follow-up for up to 5 years after EVAR, which we believe is justified for these patients.

**Figure 6.**
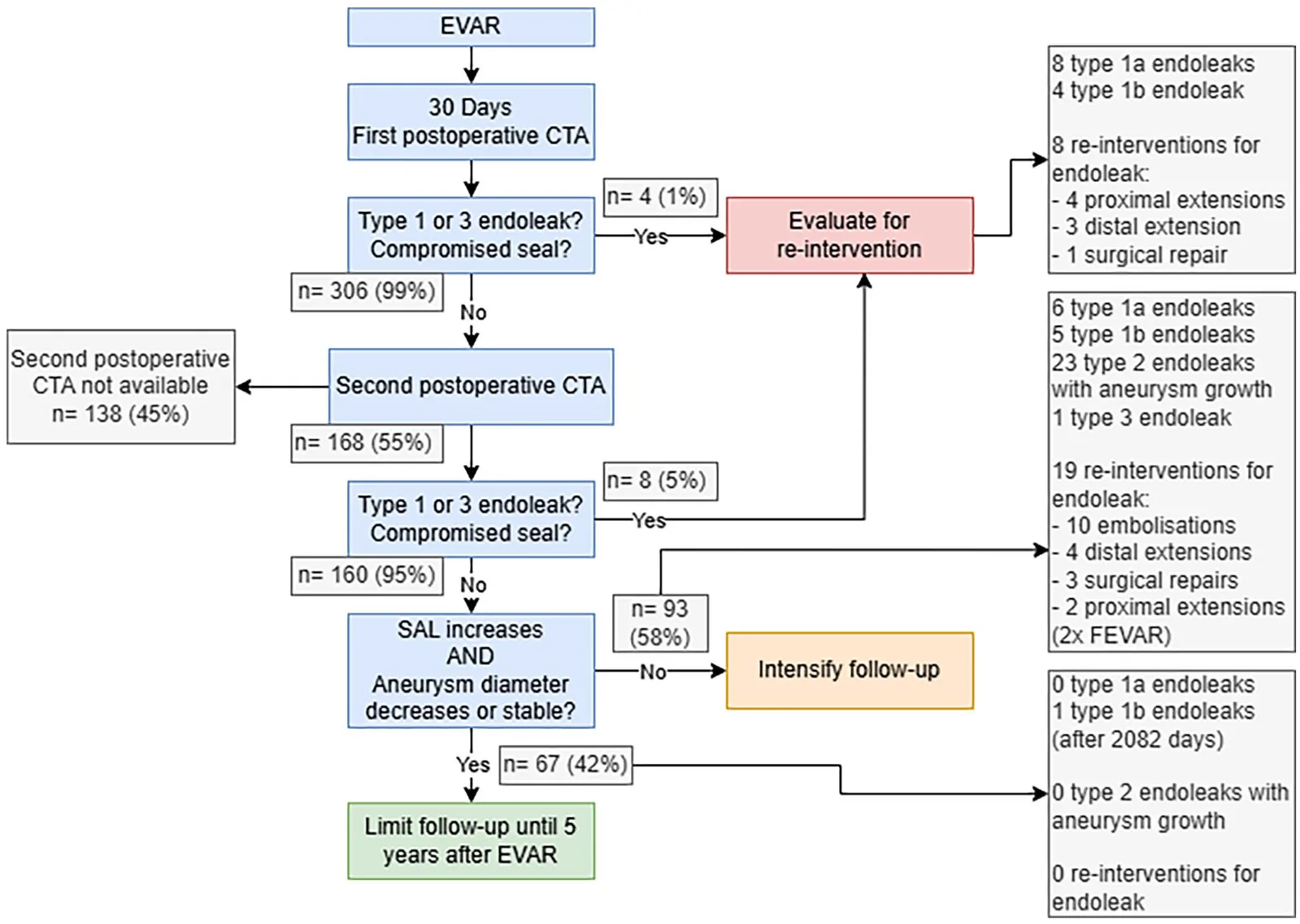
Proposed follow-up algorithm, filled in with patients of the current studied cohort. CTA, computed tomography angiography; (F)EVAR, (fenestrated) endovascular aortic repair; SAL, shortest apposition length.

We tested this new follow-up algorithm with our study cohort (Figure 6) and identified 67 patients as low-risk for developing a future endoleak. None of these patients developed type 1a endoleak or type 2 endoleak with aneurysm growth during a median follow-up of 63 (49, 79) months. Only 1 patient developed type 1b endoleak, which was seen on a CTA after 68 months. This group of patients could have benefitted from reduced imaging follow-up.

We identified 93 patients as being at higher risk for endoleak. Of these patients, 35 (38%) developed a type 1 or 3 endoleak, or a type 2 endoleak with aneurysm growth during a median follow-up of 58 (32, 72) months, 19 of 93 (20%) received secondary intervention for endoleak. This group of patients could potentially have benefited from intensified imaging follow-up.

The main strength of the current study is the use of a multicentre consecutive EVAR patient database. Patients were treated with commonly used endografts from different manufacturers. By doing this, we limited selection bias and made the results more generalizable. The downside is the lack of a fixed follow-up algorithm after EVAR. This creates considerable variation in imaging method, intensity, and timing, which creates a detection bias. It is probable that patients in the group with >1 postoperative CTA available are more likely to have a complication than patients in the group without a second postoperative CTA. Furthermore, our study was limited by the relatively low number of patients with >1 postoperative CTA available. A prospective study set up with a fixed CT follow-up regimen would tackle this problem. This would, however, be time intensive, costly, and potentially ethically not feasible due to unnecessary radiation exposure and risk of contrast nephropathy.

In our proposed algorithm, we chose a categorical variable for change of SAL (increase, stable or decrease over time) because its interpretation is simple and intuitive. However, the exact extent of change of SAL was not considered. Furthermore, the method used to measure SAL could be subject to measurement error, and while SAL represents an absolute measurement of the achieved apposition, the risk of seal failure can still be influenced by neck characteristics, the selected device, and its (over)sizing. Careful interpretation by the treating physician is required when assessing SAL and its change over time.

Another limitation of our study is that we focused on (change of) seal length in the proximal sealing zone only; the distal sealing zone was not considered. The results from our proposed follow-up algorithm suggest an association between decreasing SAL and all types of endoleak, not only type 1a endoleak. These cases likely represent a reverse correlation where decreasing SAL would be secondary to aneurysm sac expansion.

A final limitation is the use of proprietary postprocessing software to measure SAL, which is not universally available. Proximal seal measurement over centreline reconstruction would be the next best option.

## Conclusion

In this retrospective study, an increase of SAL after EVAR for infrarenal degenerative AAA was an indicator of durable success without type 1a endoleak and secondary interventions for all types of endoleak within 5 years. Decreasing SAL is associated with development of type 1a endoleak. Evaluation of the proximal seal length and its change through time could be a valuable part of follow-up after EVAR. Implementation could improve follow-up algorithms to better stratify patients at risk of endoleak.
